# Patchoulol Production with Metabolically Engineered *Corynebacterium glutamicum*

**DOI:** 10.3390/genes9040219

**Published:** 2018-04-17

**Authors:** Nadja A. Henke, Julian Wichmann, Thomas Baier, Jonas Frohwitter, Kyle J. Lauersen, Joe M. Risse, Petra Peters-Wendisch, Olaf Kruse, Volker F. Wendisch

**Affiliations:** 1 Genetics of Prokaryotes, Faculty of Biology & CeBiTec, Bielefeld University, D-33615 Bielefeld, Germany; n.henke@uni-bielefeld.de (N.A.H.); jonas.frohwitter@uni-bielefeld.de (J.F.); petra.peters-wendisch@uni-bielefeld.de (P.P.-W.); 2 Algae Biotechnology & Bioenergy, Faculty of Biology & CeBiTec, Bielefeld University, D-33615 Bielefeld, Germany; julian.wichmann@uni-bielefeld.de (J.W.); thomas.baier@uni-bielefeld.de (T.B.); kyle.lauersen@uni-bielefeld.de (K.J.L.); olaf.kruse@uni-bielefeld.de (O.K.); 3 Fermentation Technology, Technical Faculty & CeBiTec, Bielefeld University, D-33615 Bielefeld, Germany; jrisse@uni-bielefeld.de

**Keywords:** patchoulol, *Corynebacterium glutamicum*, sesquiterpene, metabolic engineering, algae, *Escherichia coli*

## Abstract

Patchoulol is a sesquiterpene alcohol and an important natural product for the perfume industry. *Corynebacterium glutamicum* is the prominent host for the fermentative production of amino acids with an average annual production volume of ~6 million tons. Due to its robustness and well established large-scale fermentation, *C. glutamicum* has been engineered for the production of a number of value-added compounds including terpenoids. Both C40 and C50 carotenoids, including the industrially relevant astaxanthin, and short-chain terpenes such as the sesquiterpene valencene can be produced with this organism. In this study, systematic metabolic engineering enabled construction of a patchoulol producing *C. glutamicum* strain by applying the following strategies: (i) construction of a farnesyl pyrophosphate-producing platform strain by combining genomic deletions with heterologous expression of *ispA* from *Escherichia coli*; (ii) prevention of carotenoid-like byproduct formation; (iii) overproduction of limiting enzymes from the 2-c-methyl-d-erythritol 4-phosphate (MEP)-pathway to increase precursor supply; and (iv) heterologous expression of the plant patchoulol synthase gene *Pc*PS from *Pogostemon cablin*. Additionally, a proof of principle liter-scale fermentation with a two-phase organic overlay-culture medium system for terpenoid capture was performed. To the best of our knowledge, the patchoulol titers demonstrated here are the highest reported to date with up to 60 mg L^−1^ and volumetric productivities of up to 18 mg L^−1^ d^−1^.

## 1. Introduction

Terpenoids are a diverse group of natural compounds that can be found in all organisms with numerous roles including, but not limited to, signaling, communication, defense, electron transfer, membrane fluidity, and pigmentation [1]. Terpenoids, also known as isoprenoids, are composed of five carbon (C5) units of isopentenyl pyrophosphate (IPP) and its isomer dimethylallyl pyrophosphate (DMAPP). IPP and DMAPP are produced by either the 2-c-methyl-d-erythritol 4-phosphate (MEP) or the mevalonate (MVA) pathways. Both pathways are capable of producing the same IPP and DMAPP precursors and the MEP pathway is found in most bacteria as well as plant plastids, while the MVA pathway is found commonly in eukaryotes and archaea [1,2]. These building blocks are condensed by prenyltransferases to form larger carbon backbones (C_10_–C_n_) that can be formed into the vast array of terpenoid molecules via the modular enzymatic reactions of the terpene synthases. Terpene backbones can also be functionalized by further down-stream enzymes such as cytochrome P450s, yielding the observed array of chemical diversity of terpenoid natural compounds [3].

The demand for naturally produced terpenoids is increasing due to increased consumer awareness and growing demand for naturally sourced materials in everyday products such as food, cosmetics, perfumes, and household chemicals. Although some terpenoids can be extracted from plant material, terpenoid production processes using bio-based microbial platforms are gaining attention as sustainable and economical alternatives. Terpenoid biosynthesis is inherently modular, with defined enzymatic steps leading from precursors to specific terpenoid products [3]. As all organisms contain the building blocks for terpenoid biosynthesis, it is possible to selectively transfer terpene synthases from organisms that naturally produce a desired compound into biotechnologically amenable hosts. Controlled microbial fermentation and production of terpenoids can have numerous benefits over harvesting and extraction from native organisms including containment, scalability, reliable product yield, shorter generation times, and minimizing environmental impacts from harvesting or agricultural cultivation.

Farnesyl pyrophosphate (FPP), the prenylated precursor of sesquiterpenoids (C15), is formed by condensation of DMAPP and two molecules of IPP by the enzyme farnesyl pyrophosphate synthase (FPPs). Sesquiterpenoid products are generated from FPP by sesquiterpene synthases. The sesquiterpenoid alcohol patchoulol is harvested from the leaves of the plant *Pogostemon cablin*, and is used as a component in perfumes, incense, and natural insect repellents [4]. *P. cablin* is traditionally cultivated in Indonesia and India where steam distillation of leaves is used to extract its essential oil, the main odor compound in this oil is the sesquiterpenoid alcohol patchoulol [5,6]. Patchouli oil is currently produced via traditional agriculture and steam distillation, 2.2–2.8 kg of patchouli oil can be extracted from 100 kg of dried patchouli leaves. This process lasts 8 hours and can consume 40 liters of kerosene [6]. As this process is energy and resource intensive, microbial fermentative processes for the production of patchoulol could minimize its environmental footprint and improve product yields. The conversion of FPP to patchoulol is mediated by a single enzyme, the patchoulol synthase (*Pc*PS; PTS, uniprot: Q49SP3) [5,7], and heterologous expression of this enzyme has been used to produce this fragrance compound in several microbial hosts including *Saccharomyces cerevisiae* [8], the moss *Physcomitrella patens* [9] and the green microalga *Chlamydomonas reinhardtii* [10]. Mixtures of sesquiterpenes and alcohols present in patchouli oil obtained by fermentation have been marketed (e.g., by Firmenich). It was shown that overproduction of the patchoulol synthase in *Escherichia coli* produced several sequiterpenes besides patchoulol [5]. Thus, this product promiscuity could find suitable applications in protein engineering towards other sequiterpenes and the specificity for production of patchoulol itself could be a target for further enzyme engineering studies for the *Pc*PS. 

The Gram-positive bacterium *Corynebacterium glutamicum* is the workhorse of industrial biotechnology for amino acid production and is already cultivated in industrial processes for the production of l-glutamate and l-lysine. Fermentations with this host are characterized by robust growth, high cell densities and high volumetric productivities [11,12,13]. The annual production volume of l-glutamate and l-lysine in industrial processes with *C. glutamicum* is estimated to reach 6 million tons in 2023 [14]. Currently, industrial production plants for *C. glutamicum* cultivation are found worldwide indicating that engineering of this organism for expanded profiles of natural products has great biotechnological and commercial value potentials. *C. glutamicum* is not only a natural l-glutamate producer, but also accumulates a yellow carotenoid, decaprenoxanthin, in its membranes [15,16]. The carotenoid biosynthesis of *C. glutamicum* has been analyzed in more detail over the last years [15]. Based on this knowledge, *C. glutamicum* has been engineered for production of a range of high value terpenoids including short-chain terpenoids [17,18] and various C40 and C50 carotenoids [19,20]. Besides gene deletion, integration and overexpression, also other methodologies such as regulator engineering [21], general transcription machinery engineering [22] and on-demand gene expression with photocaged-IPTG [18] were successfully demonstrated in this host to develop terpenoid overproducing strains. Furthermore, coproduction of the terpenoid astaxanthin and the amino acid l-lysine by a single *C. glutamicum* strain was established [23]. The coproduction of two value-added compounds represents a novel fermentation strategy in which a biomass-bound and a secreted substance were simultaneously produced during a single fermentation run. This example indicates the great volumetric productivity potential of engineered *C. glutamicum* for feed market applications [23].

In this work, we seek to expand the product profile range of *C. glutamicum* by engineering this organism for the production of the sesquiterpenoid perfume product patchoulol. Here, patchoulol production was achieved by combining construction of an FPP-producing platform strain, wherein carotenoid-like byproduct formation was prevented, with overproduction of rate-limiting MEP pathway enzymes. This strain was used for heterologous expression of the *P. cablin* patchoulol synthase (*Pc*PS) and patchoulol production as well as a proof-of-principle liter-scale bioreactor fermentation with product capture using a two-phase organic solvent-culture overlay were demonstrated.

## 2. Materials and Methods

### 2.1. Bacterial Strains, Media and Growth Conditions 

The strains and plasmids used in this work are listed in Table 1. *C. glutamicum* ATCC 13032 [24] was used as the parental strain for all metabolic engineering approaches. Pre-cultivation of *C. glutamicum* strains was performed in BHI/LB medium (Carl Roth, Karlsruhe, Germany) with 50 mM glucose. For main cultivation in CGXII minimal medium [25], these pre-cultivated cells were washed once with CGXII medium without carbon source and inoculated to an initial *OD*_600_ of 1. 100 mM glucose was added as carbon and energy source. Standard cultivations of *C. glutamicum* were performed at 30 °C in a volume of 20 mL in 100 mL flasks without baffles shaking at 120 rpm. The *OD*_600_ was measured in dilutions using a Shimadzu UV-1202 spectrophotometer (Duisburg, Germany). For capturing of the volatile patchoulol 10% (*v*/*v*) of dodecane was added to the main culture [17]. *E. coli* DH5α was used as host for all cloning aspects and cultivated in LB medium at 37 °C overnight. When appropriate, tetracyclin, spectinomycin or kanamycin were added in concentrations of 5, 100 and 25 µg mL^−1^, respectively. Gene expression was induced by addition of 1 mM IPTG to the main culture.

### 2.2. Recombinant DNA Work

Plasmids were constructed with Gibson assembly [32] or classical restriction and ligation. PCR-generated fragments (All-in HiFi, highQu, Kraichtal, Germany) were assembled into isolated plasmids (Plasmid GeneJET Miniprep kit, Thermo Fisher Scientific, Schwerte, Germany) that were linearized by restriction. Oligonucleotides used in this study were obtained from Metabion (Planegg/Steinkirchen, Germany) and are listed in Table 2. Standard reactions like restriction, and PCR were performed as described previously [33]. If applicable, PCR products were purified using the PCR clean-up and gel extraction kit (Macherey-Nagel, Düren, Germany). For transformation of *E. coli* DH5α, the RbCl method was used and *C. glutamicum* was transformed via electroporation [34] at 2.5 kV, 200 Ω, and 25 µF. Vector construction was confirmed by sequencing of cloned inserts.

### 2.3. Deletion and Exchange Mutagenesis in the Genome of Corynebacterium glutamicum

For targeted deletion of the carotenogenic operons (cg0717-cg0723 and cg2668-cg2672), *crtE* (cg0723) and *idsA* (cg2384), the suicide vector pK19*mobsacB* was used [31]. Genomic regions flanking the corresponding genes/operons were amplified from genomic DNA of *C. glutamicum* WT using primer pairs A/B and C/D (Table 2), respectively. The resulting amplificates were cloned into pK19*mobsacB* via Gibson method resulting in the construction of deletion vectors (Table 1). Deletions were carried out via two-step homologous recombination as well as the selection for the first and second recombination events were carried out as described previously [25]. Successful removals of genes or operons were verified by PCR analysis of the constructed mutants using primer pair E/F (Table 2).

### 2.4. Fermentation of Corynebacterium

A bioreactor with a total volume of 3.7 L and a working volume of 2.0 L was used for batch and fed-batch fermentations (KLF, Bioengineering AG, Switzerland). The stirrer axis was equipped with three six-bladed rushton turbines in a distance of 6 cm, 12 cm, and 18 cm from the bottom. The relative dissolved oxygen saturation in the medium (*rDOS*) (Mettler-Toledo, Greifensee, Switzerland) and pH (Hamilton, Switzerland) were monitored with electrodes. The pH was kept at 7.0 by automated addition of KOH 4 M and 10% (*w*/*w*) phosphoric acid. The initial volume of the fermentations was 2 L. For feeding approximately 730 mL volume was added, depending on the *rDOS*. Fermentations were performed with 0.2 bar overpressure and an aeration rate of 2 NL min^−1^. The initial stirrer frequency was set to 200 min^−1^ and speed was increased in steps of 2% when *rDOS* profile felt below 30% (fed-batch) or 60% (batch), respectively. 

Struktol^®^ was added manually as an antifoam reagent. For the fed-batch procedure the feeding profile was activated when the *rDOS* was about 60% and stopped when *rDOS* felt below 60%. Samples were taken regularly and were stored at 4 °C until further use. Plasmid-driven gene expression was induced in the exponential growth phase (t = 5 h). Capturing of patchoulol was conducted through a dodecane overlay that was applied 5 (batch) or 25 (fed-batch) hours after inoculation. 

The fermenters were inoculated with a fresh overnight culture grown at 30 °C and 120 rpm (amplitude: 2.5 cm) to an initial *OD* of ~1. The preculture medium contained: 13.6 g L^−1^ soypeptone, 7 g L^−1^ yeast extract, 2.5 g L^−1^ NaCl, 2.3 g L^−1^ K_2_HPO_4_, 1.5 g L^−1^ KH_2_PO_4_, 0.14 g L^−1^ MgSO_4_·H_2_O, 40 g L^−1^ d-glucose monohydrate, 25 mg L^−1^ tetracycline, and 25 mg L^−1^ kanamycin. The fermentation was performed on the basis of the same medium. Feeding medium contained 300 g L^−1^ d-glucose monohydrate and 75 g L^−1^ yeast extract.

### 2.5. Patchoulol Capture and Quantification

The clean dodecane supernatants were analyzed via GC–MS using a TraceGC gas chromatograph (Thermo Scientific, Waltham, MA, USA) and ITQ ion trap mass spectrometer (Thermo Scientific, Waltham, Massachusetts, USA equipped with a AS 3000 autosampler (Thermo Scientific, Schwerte, Germany). As a column system a 30 m × 0.25 mm VF-5 ms column coated with 0.25 µm of 5% diphenyl and 95% dimethylsiloxane (Varian GmbH, Darmstadt, Germany) was used. Temperature profile was set as the following: injector (250 °C), interface (250 °C) and ion source (220 °C). 1 µL of sample was injected in splitless mode. A constant flow of 1 mL min^−1^ helium was used as a carrier gas. The oven temperature profile was set as the following: 80 °C for one minute, then raised to 120 °C at 10 °C min^−1^, followed by 3 °C min^−1^ to 160 °C, and further to 270 °C at 10 °C min^−1^, which was held for 2 min. Mass spectra were recorded after the dodecane peak eluted (12 min) using a scanning range of 50–750 *m*/*z* at 20 scans s^−1^. Chromatograms were evaluated with Xcalibur software version 2.0.7 (Thermo Scientific, Germany). The NIST 05 library (National Institute of Standards and Technology, Gaithersburg, MD; ThermoFinnigan) was used to identify substances for which no standard was available. Standard calibration curves in the range of 1–450 µM (or 0.5–100 mg L*^−^*^1^) patchoulol in dodecane were used to quantify the amount of patchoulol (R^2^ ≈ 0.99). 250 µM α-humulene was applied in each sample as internal standard. Extracted-ion chromatograms (XIC) with mass ranges of 93.00 (α-humulene), 138.50 and 222.00 (patchoulol) were used (see Figure S2).

## 3. Results

### 3.1. Patchoulol Production in Shake Flasks with Metabolically Engineered Corynebacterium glutamicum

**Figure 1:**
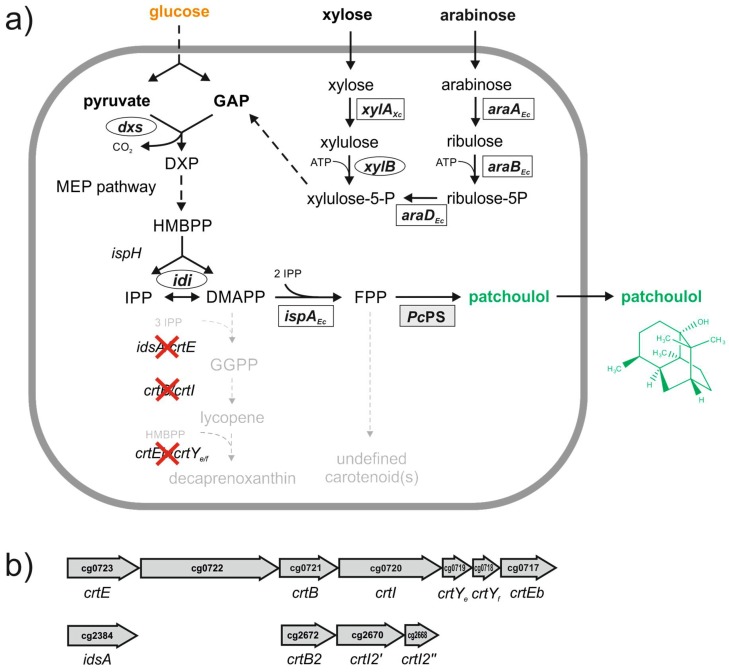
Patchoulol production by engineered *Corynebacterium glutamicum*. (**a**) Schematic representation of the patchoulol pathway in recombinant *C. glutamicum*. Overexpressed genes are in bold, endogenous genes are in circles, heterologous genes are boxed (clear background for bacterial origin and shaded background for eukaryotic origin), genes deletions are indicated by red crosses, gene names are indicated next to the reactions they catalyze, single reaction are represented by arrows, pathways with more than one reaction by dashed arrows. Abbreviations: *dxs*, 1-deoxy-d-xylulose-5-phosphate synthase; *ispH*, 4-hydroxy-3-methylbut-2-en-1-yl diphosphate reductase; *idi*, isopentenyl-diphosphate isomerase; *idsA*, geranylgeranyl diphosphate synthase; *crtE*, geranylgeranyl diphosphate synthase; *crtB*, phytoene synthase; *crtI*, phytoene desaturase; *crtEb*, lycopene elongase; *crtY_e/f_*, lycopene β-cyclase; *xylB*, xylulokinase; *ispA_Ec_*, farnesyl pyrophosphate synthase from *Escherichia coli*; *Pc*PS, plant patchoulol synthase from *Pogostemon cablin*; *xylA_Xc_*, xylose isomerase from *Xanthomonas campestris*; *araA_EC_*, arabinose isomerase from *E. coli*; *araB_EC_*, ribulokinase from *E. coli*; *araD_EC_*, ribulose-5-phosphate-4-epimerase from *E. coli*; GAP, glycerol aldehyde phosphate; DXP, 1-deoxy-d-xylulose-5-phosphate; HMBPP, 4-hydroxy-3-methylbut-2-enyl diphosphate; IPP, isopentenyl pyrophosphate; DMAPP, dimethylallyl pyrophosphate; FPP, farnesyl pyrophosphate; GGPP, geranylgeranyl pyrophosphate; (**b**) Carotenogenic genes and operons in *C. glutamicum*. The major crt operon contains: *crtE* (cg0723), geranylgeranyl diphosphate synthase; (cg0722); *crtB* (cg0721), phytoene synthase; *crtI* (cg0720), phytoene desaturase; *crtY_e_* (cg0719); *crtY_f_* (cg0718); *crtEb* (cg0717), phytoene synthase. The small crt operon contains: *crtB2* (cg2672), phytoene synthase; *crtI2′* (cg2670), non-functional phytoene desaturase; *crtI2″* (cg2668), non-functional phytoene desaturase. *idsA* (cg2384), geranylgeranyl diphosphate synthase.

*C. glutamicum* possesses two genes encoding GGPP synthases (Figure 1) with *idsA* encoding the major GGPPS. Their biochemical characteristics and mutant studies revealed that primarily GGPP is formed by *idsA* and *crtE* [35]. Therefore, the deletion mutant Δ*crtE*Δ*idsA* derived from *C. glutamicum* wild type ATCC 13032 that was already employed for successful production of short-chain terpenoids [17,18] was used here as the base strain for patchoulol production (Table 3). 

The deletion of both GGPP synthases genes *idsA* and *crtE* results in the accumulation of the central terpenoid precursor molecules IPP and DMAPP due to the lack of other short-chain prenyltransferases in *C. glutamicum*. Heterologous overexpression of *ispA* from *E. coli* coding for FPP synthase and overexpression of a codon-optimized *Pc*PS encoding the patchoulol synthase from *P. cablin* (Uniprot Q49SP3) in this strain enabled patchoulol production from glucose (PAT1) (Figure 1). PAT1 cells even exhibited a distinct earthy odor of the patchouli alcohol under IPTG induction on the agar plate. Cultivation of PAT1 in 20 mL CGXII with 100 mM glucose produced 0.20 ± 0.03 mg L^−1^ patchoulol (concentrations are given based on the total culture medium volume) within 48 h incubation. 1 mM IPTG was added to the culture medium in the exponential growth phase (t = 5 h) in order to induce plasmid-driven gene expression of the prenyltransferase and terpene synthase genes. At the same time, 2 mL of dodecane was added as an overlay to capture the volatile product. Interestingly, *C. glutamicum* PAT1 cells exhibited a colored phenotype. The pigmentation may be due to the formation of (a) C30 carotenoid-like structure(s) under the assumption that endogenous carotenogenic enzymes accept FPP as substrate in the absence of GGPP (Figure 1). Thus, deletion of all carotenogenic genes from the *C. glutamicum* genome was conducted in order to prevent formation of undesired terpenoid byproducts (see Figure S1). As expected, the corresponding strain PAT2 lacking both crt operons [15] as well as the major GGPP synthase [35] showed the desired white phenotype indicating that carotenoid-like byproducts were no longer accumulated (Figure 1 and Figure S1). The patchoulol titer of PAT2 (0.22 ± 0.02 mg L^−1^) remained comparable to that produced by PAT1 (Table 3). 

As known from production of other short- and long-chain terpenoids by recombinant *C. glutamicum* [18,36], precursor supply was expected to limit patchoulol production and therefore was set as a target for further engineering. Overproduction of the first enzyme of the MEP-pathway, Dxs, as well as the isomerase Idi, have been shown to be highly effective for improved terpenoid production both in *C. glutamicum* [17,36] and *E. coli* [37,38] due to the generation of increased precursor supply for terpenoid products. Here, plasmid-driven overexpression of endogenous *dxs* and *idi*improved patchoulol production approximately 2-fold (Table 3). The strain PAT3 produced 0.46 ± 0.07 mg L^−1^ patchoulol in shake flask cultures within 48 h. The volumetric productivity of 0.23 ± 0.03 mg L^−1^ d^−1^ is among the highest reported for patchoulol production [10].

### 3.2. Patchoulol Production from Alternative Carbon Sources

In order to test if patchoulol production from *C. glutamicum* could be conducted in a sustainable manner on carbon sources which do not compete for resources with food and feed production, small scale fermentations of PAT3 were carried out with the pentoses l-arabinose and d-xylose. These pentoses can be derived from agricultural waste products such as wheat or rice straw hydrolysates [29,39]. Since the PAT3 strain is not able to grow on either substrate, metabolic engineering for utilization of arabinose and xylose was performed. First, plasmid-driven overproduction of arabinose isomerase, ribulokinase and ribulose-5-phosphate-4-epimerase encoded by the *araBAD* operon from *E. coli* enabled *C. glutamicum* growth and the production of patchoulol. These enzymes convert arabinose to xylulose 5-phosphate, a central intermediate of the pentose phosphate pathway. Second, plasmid-driven overproduction of xylose isomerase and xylulokinase encoded by *xylA* from *X. campestris* and *xylB* from *C. glutamicum*, respectively, allowed phosphorylation and isomerization of the pentose sugar to xylulose 5-phosphate [29]. 

**Figure 2:**
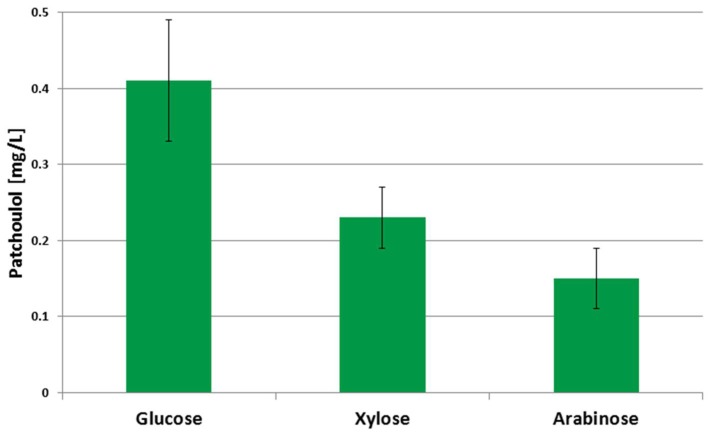
Patchoulol production from alternative carbon sources in 20 mL shake flasks after 48 h. Patchoulol titers from 10 g L^−1^ of glucose, xylose, and arabinose are shown as mean and arithmetic deviation from the mean from biological duplicates. Cultivation was performed in CGXII minimal medium with 1 mM IPTG and 10% (*v*/*v*) of dodecane.

As shown in Figure 2 patchoulol production was possible from the cultivation of engineered *C. glutamicum* on the pentoses arabinose and xylose. The control strain PAT3 (pEKEx3) produced 0.41 ± 0.08 mg L^−1^ patchoulol from 10 g L^−1^ glucose. The strains PAT3 (pEKEx3_*araBAD*) and PAT3 (pEKEx3_*xylAB*) produced 0.15 ± 0.04 mg L^−1^ and 0.23 ± mg L^−1^ patchoulol on the basis of 10 g L^−1^ of the respective pentose sugar. The observed differences in patchoulol titers may reflect that glucose can be catabolized in glycolysis and/or the oxidative pentose phosphate pathway while arabinose and xylose catabolism proceeds via the oxidative pentose pathway. To comprehend the differences of growth and production between the substrates glucose, arabinose, and xylose a full carbon-13 based labelling flux metabolic analysis is needed, however, the data depicted in Figure 2 provide a proof-of-concept that patchoulol production from xylose and arabinose is possible.

### 3.3. Batch Fermentation for Patchoulol Production

**Figure 3:**
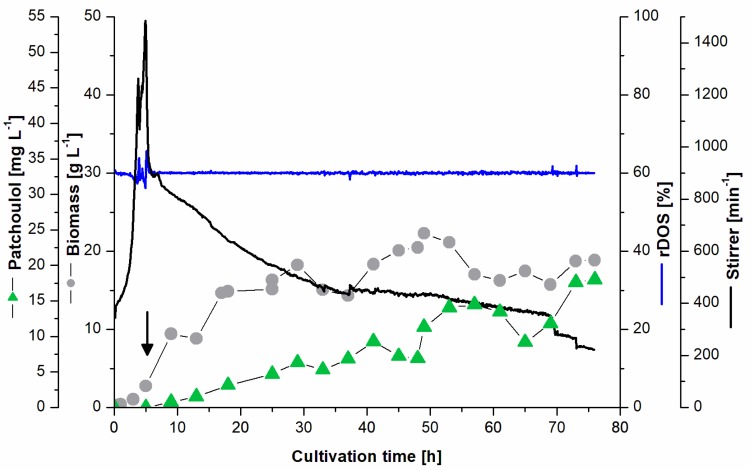
Batch fermentation for patchoulol production. PAT3 was cultivated in batch mode containing *i*.a. 40 g L^−1^ glucose monohydrate over 80 h. Patchoulol titer is indicated with green triangles (mg L^−1^); biomass concentration (CDW) is indicated in grey circles (g L^−1^), relative dissolved oxygen saturation is indicated in blue (*rDOS* %) and the stirrer frequency is shown in black (min^−1^).

Since the volumetric patchoulol productivity of PAT3 was among the highest reported [10], patchoulol production was tested in bioreactors in order to investigate stability and reliability of the process. First, PAT3 was cultivated in a 2 L fermenter in batch mode with 40 g L^−1^ of glucose monohydrate. Product concentration was monitored over time (Figure 3). The relative dissolved oxygen saturation (*rDOS*) was kept constant at 60% by regulation of the rotary frequency of the stirrer. A maximal titer of 18 mg L^−1^ patchoulol (t = 76 h) was achieved in the two-phase culture system containing 2 L culture and 200 mL dodecane overlay when dodecane and 1 mM IPTG were added in the early exponential growth phase 5 h after inoculation (at *OD* ~10). Biomass concentration (cell dry weight) was calculated on the basis of the optical density (600 nm). Measurements of the optical density and corresponding biomass were adjusted after the addition of dodecane due to emulsification. The maximal volumetric productivity was reached after 53 h with around 6.4 mg L^−1^ d^−1^, corresponding a current titer of ~14 mg L^−1^ patchoulol in the culture medium (Figure 3). The patchoulol titer represents an improvement by a factor ~30 in comparison to the shake flasks experiments in 20 mL scale.

### 3.4. Fed-Batch Fermentation for Patchoulol Production

**Figure 4:**
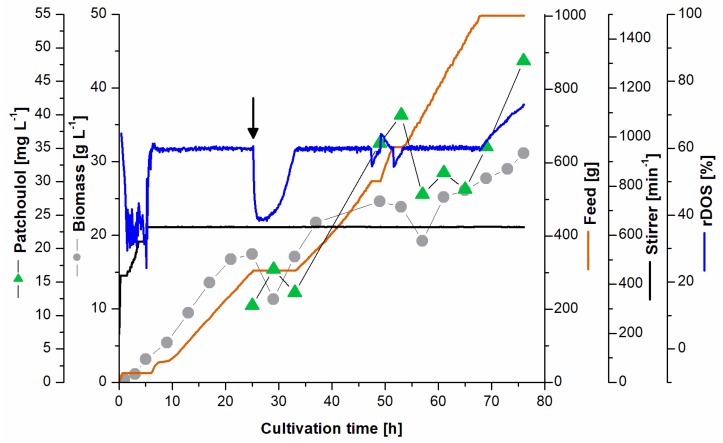
Fed-Batch fermentation for patchoulol production. PAT3 was cultivated in fed-batch mode containing i.a. 40 g L^−1^ glucose monohydrate over 80 h. Patchoulol titer is indicated with green triangles (mg L^−1^); biomass concentration (CDW) in grey circles (g L^−1^), feed in orange (g of medium), relative dissolved oxygen saturation is indicated in blue (*rDOS* %), and the stirrer frequency is shown in black (min^−1^).

Batch fermentation here resulted in a 30-fold improved patchoulol titer over shake flask experiments, which prompted us to investigate fed-batch mode fermentation with PAT3 in a 2 L fermenter. The batch phase was performed as described previously in complex medium supplemented with 40 g L^−1^ glucose monohydrate as primary carbon and energy source. Plasmid-born gene expression was induced by the addition of 1 mM IPTG after 5 h of cultivation in order to prevent accumulation of the toxic precursor molecules DMAPP and IPP. Dodecane was added in the feeding phase, 25 h after inoculation at an *OD* ~75 to capture the volatile patchoulol. The feeding solution contained 300 g L^−1^ glucose monohydrate and yeast extract as nitrogen source. Feeding was controlled via the *rDOS* profile and started ~10 h after inoculation when the *rDOS* reached 60%. A total feed amount of ~1000 g medium (~220 g glucose) was applied over the whole process. As a result, a high biomass titer of ~30 g L^−1^ CDW was reached (Figure 4). The patchoulol titer of ~50 mg L^−1^ (in relation to the culture volume) was reached around 76 h after inoculation, corresponding to a volumetric productivity of ~15 mg L^−1^ d^−1^. The maximal volumetric productivity of ~18 mg L^−1^ d^−1^ was reached in the feeding phase of the two-phase culture system (t = 53 h). The titer of patchoulol in the culture medium reached 60 mg L^−1^ at 142 h of fermentation (data not shown). 

## 4. Discussion

In this work, patchoulol production by *C. glutamicum,* the microbial cell factory for the million-ton-scale production of amino acids [14], was demonstrated (see Figure S2). Strain engineering addressed heterologous overproduction of FPP synthase, overproduction of codon-optimized plant patchoulol synthase (*Pc*PS), prevention of terpenoid byproducts, increased flux through the MEP-pathway and balancing of the DMAPP to IPP ratio. To the best of our knowledge, the titer of 60 mg L^−1^ and the volumetric productivity of 18 mg L^−1^ d^−1^ achieved in 2 L bioreactor cultures are the highest patchoulol yields reported to date for microbial fermentation. For comparison, the highest reported titers and volumetric productivities obtained with phototrophic hosts were ~1 mg L^−1^ and ~0.23 mg L^−1^ d^−1^, respectively, [10]. 

First, heterologous overproduction of FPP synthase and the plant patchoulol synthase were combined in PAT1, which resulted in the formation of patchoulol. However, overproduction of FPP in the presence of endogenous carotenogenic enzymes CrtB and CrtI, but in the absence of their cognate substrate GGPP, resulted in the accumulation of a carotenoid-like pigment indicating the native enzymes have some affinity for the produced FPP. It is conceivable that C30 carotenoids, for example, 4,4′-diapolycopene have been formed. Thus, deletion of the carotenogenic operons and genes in the wild-type background strain were performed to avoid accumulation of untargeted terpenoid by-products. Although deletion of the carotenogenic genes did not improve patchoulol production, the white cells of PAT2 and PAT3 may be favorable for patchoulol downstream processing. Usually, clear products are preferred for cosmetic ingredients and extraction of colored terpenoid pigment by-products can thus be avoided in these strains. 

Patchoulol production could be improved by 2-fold due to an improved flux through the MEP-pathway, mediated by the overexpression of *dxs*, and an improved balance of the precursors DMAPP and IPP was achieved by the overexpression of *idi*. The strain PAT3 produced ~0.5 mg L^−1^ patchoulol in 20 mL scale from 100 mM glucose. The MEP-pathway is supposed to be heavily regulated at many levels [1,40] and overexpression of both *dxs* and *idi* were shown to be beneficial for improving terpenoid production from several organisms, including *E. coli* and *C. glutamicum*. Indeed, *dxs* and *idi*, are considered the main rate-limiting steps within the MEP pathway [37,41]. While overproduction of Dxs enhances the flow through the MEP pathway to ultimately produce more IPP and DMAPP, the cause for the beneficial role of Idi overexpression, and its effect on IPP and DMAPP ratios, is still under investigation. In *E. coli*, overproduction of Idi led to a shift of the natural IPP and DMAPP ratio of 5:1 [42] towards DMAPP, thus allowing for the production of more FPP since it is made by two molecules of IPP and one molecule of DMAPP [37].

Production titers of the sesquiterpene (+)-valencene in *C. glutamicum* were shown to be improved by the use of caged-IPTG, allowing photo-induced production onset with UV light, compared to conventional IPTG in microtiter-scale production experiments [18]. Whether this effect can be transferred to patchoulol production in flask or even bioreactor scale experiments needs to be evaluated. However, the relatively high price of caged-IPTG compared to IPTG, makes utilization in larger scales not feasible [18]. 

After establishment of patchoulol production from *C. glutamicum* in small scale (20 mL), scale up was conducted. Here, the 100-fold increase in scale from 20 mL to 2 L was successfully performed in a bioreactor. A 30-fold increased patchoulol titer in comparison to shake flasks was observed. The strong improvement is likely due to improved oxygen input and saturation in the bioreactor compared to shake flasks and/or the consistency of pH in the controlled reactor. A beneficial effect due to improved dodecane mixing with the microbial culture is also a likely factor in the observed product titers. Dodecane captures volatile hydrophobic compounds and also supports the release or extraction of these compounds from hydrophobic biological membranes [43]. In the fed-batch fermentation, a patchoulol titer of ~60 mg L^−1^ was achieved within 142 h of cultivation, corresponding to a 130-fold increase in productivity in comparison to the small-scale flask fermentation and an about 4-fold increase compared to the batch-mode bioreactor culture. The significant drop in the relative dissolved oxygen (*rDOS*) at 25 h can be explained with coalescence due to dodecane application. With a volumetric productivity of ~18 mg L^−1^ d^−1^ the production process presented here is superior to other production systems in respect to maximal titers and volumetric productivities. Both batch and fed-batch fermentations conducted here had maximal volumetric productivities around 53 h after inoculation, with 6.4 and 18 mg L^−1^ d^−1^, respectively. In this work, patchoulol was captured in a dodecane phase, allowing a first separation of the product from the cell fraction. Dodecane as a linear petrochemical hydrocarbon is considered a green solvent from the cluster 2 of the chemometric solvent selection guide with a high confidence level [44]. Alternatively, patchoulol capture is possible with isopropyl myristate, which is classified as a green solvent [45]. Both in batch and fed-batch fermentations, plasmid expression was induced 5 h after inoculation. Since the strain PAT3 overproduces the terpenoid precursor molecules DMAPP and IPP, accumulation of these toxic compounds might affect the cells negatively as has been shown for *Bacillus subtilis* and *E. coli* [46]. 

Interestingly, PAT3 cells cultivated under fed-batch mode produced additional sequiterpenes. According to the GC-MS library search, azulene and caryophyllene. However, the peak intensities of these products were much lower and product formation occurred only in the late stationary phase (data not shown). The promiscuity of the used patchoulol synthase from *P. cablin* was already mentioned in literature with a least 13 additional sequiterpene products [5]. A rough comparison of the in vitro enzymatic activity of ~0.02 unit mg^−1^, corresponding to 213,000 µg g CDW^−1^ d^−1^ [7,47] of purified *Pc*PS is in accordance with the volumetric productivity ~15 mg L^−1^ d^−1^, 1000 µg g CDW^−1^ d^−1^, of this study under the assumption that approximately 0.3% of the cellular protein of the producer strain is *Pc*PS. 

Sequiterpenes are interesting products and their heterologous production has been in established in several microorganisms. The biofuel component farnesene [48] can be produced with volumetric productivities of up to 16.9 g L^−1^ d^−1^ and a maximum titer of 104.3 g L^−1^ [49], which is one of the highest reported terpenoid titers so far. Heterologous production of the anti-malaria drug precursor artemisinic acid is a prominent example for a high pricing terpenoid that was established in both eukaryotic and prokaryotic host organisms, *S. cerevisiae* and *E. coli*, respectively. A titer of 27.4 g L^−1^ amorphadiene is one of the highest reported for *E. coli* whereas functionalization of this sesquiterpene by a plant-based P450 enzyme enabled production of 105 mg L^−1^ artemisinic acid [50]. To the best of our knowledge, artemisinic acid production reached 25 g L^−1^ with yeast, which is the highest reported to date [51]. These overproducing strains share intensive metabolic engineering, especially the expression of the de-regulated MEV-pathway, which in bacterial hosts has been demonstrated to be a powerful strategy for boosting overall terpenoid yields and could also be a suitable target for *C. glutamicum*. Establishment of this second precursor pathway might result in a significant increase of DMAPP and IPP production as the heterologous MEV-pathway is not expected to be regulated in *C. glutamicum*. In the terpenoid production strains mentioned above genes have been integrated and expressed from chromosomal locations using strong constitutive natural or synthetic promoters rather than from IPTG inducible plasmids. This increased genetic stability as well as gene expression represent a valuable strategy to further improve the patchoulol producing *C. glutamicum* strains described here. Since a number of terpenes inhibit bacterial growth further strain engineering may be guided, e.g., by RNAseq analysis of gene expression +/− patchoulol, to improve patchouli production if patchoulol inhibits growth. This may also be relevant to adjust the fermentation process. 

## 5. Conclusions

Metabolic engineering of *C. glutamicum* led, to the best of our knowledge, to the highest patchoulol titers (up 60 mg L^−1^) and volumetric productivities (up 18 mg L^−1^ d^−1^) of microbial fermentation reported to date. Production of patchoulol by recombinant *C. glutamicum* from substrates not competing for use in human or animal nutrition such as the pentose sugars l-arabinose and d-xylose was enabled. Patchoulol production from *C. glutamicum* was successfully demonstrated in batch and fed-batch bioreactor set-ups with two-phase organic solvent overlay for capture of the semi-volatile sesquiterpenoid product.

## Figures and Tables

**Table 1:** Strains and plasmids used in this study.

STRAIN; PLASMID	Relevant Characteristics	Reference
*Corynebacterium* *glutamicum* **STRAINS**		
WT	Wild type, ATCC 13032	[24]
Δ*crtE*Δ*idsA*	*crtE* (cg0723) and *idsA* (cg2384) deletion mutant of ATCC 13032	[17]
Δ*crtOP*Δ*idsA*Δ*crtB2I’I2*	*crtOP* (cg0717- cg0723), *idsA* (cg2384) and *crtB2I’I2* (cg2668-cg2672) deletion mutant of ATCC 13032	this work
PAT1	Δ*crtE*Δ*idsA* (pECXT-*ispA-PcPS*)(pEKEx3)	this work
PAT2	Δ*crtOP*Δ*idsA*Δ*crtB2I’I2* (pECXT_*ispA-PcPS*)(pVWEx1)	this work
PAT3	Δ*crtOP*Δ*idsA*Δ*crtB2I’I2* (pECXT_*ispA-PcPS*)(pVWEx1_*dxs-idi*)	this work
**OTHER STRAINS**		
*Escherichia coli* DH5α	F-*thi-*1 *endA1 hsdr17*(r-, m-) *supE44* Δ*lacU169* (Φ80*lacZ*ΔM15) *recA1 gyrA96*	[26]
**PLASMIDS**		
pOpt_*PcPS*	Shuttle vector containing *PcPS* from *Pogostemon cablin* codon-optimized for *Corynebacterium glutamicum* (gene synthesis) (Uniprot Q49SP3)	this work
pEC-XT99A (pEC-XT)	Tet^R^, *P_trc_lacI^q^*, pGA1 *oriV_Cg_*, *C. glutamicum*/*E. coli* expression shuttle vector	[27]
pEC-XT_*ispA-PcPS*	pEC-XT derivative for IPTG-inducible expression of *ispA* from *E. coli* and codon-optimized *Pc*PS from *P. cablin* (Uniprot Q49SP3) containing an artificial ribosome binding site	this work
pEKEx3	Spec^R^, *P_tac_lacI^q^*, pBL1 *oriV_Cg_*, *C. glutamicum*/*E. coli* expression shuttle vector	[28]
pEKEx3_*araBAD*	pEKEx3 derivative for IPTG-inducible expression of the *araBAD* operon from *E. coli* containing an artificial ribosome binding site	this work
pEKEx3_*xylA*B	pEKEx3 derivative for IPTG-inducible expression of *xylA* from *Xanthomonas campestris* and *xylB* from *C. glutamicum* containing an artificial ribosome binding site	[29]
pVWEx1	Km^R^, *P_tac_lacI^q^*, pHM519 *oriV_C_*_g_, *C. glutamicum*/*E. coli* expression shuttle vector	[30]
pVWEx1_*dxs-idi*	pVWEx1 derivative for IPTG-inducible expression of *dxs* (cg2083) and *idi* (cg2531) from *C. glutamicum* containing an artificial ribosome binding site	[18]
pK19*mobsac*B	Km^R^; *E. coli*/*C. glutamicum* shuttle vector for construction of insertion and deletion mutants in *C. glutamicum* (pK18 *oriV_Ec_ sacB lacZα*)	[31]
pK19*mobsacB*Δ*crtE*	pK19*mobsacB* with a crtE (cg0723) deletion construct	[17]
pK19*mobsacB*Δ*idsA*	pK19*mobsacB* with a *idsA* (cg2384) deletion construct	[17]
pK19*mobsacB*Δ*crtOP*	pK19*mobsacB* with a *crtOP* (cg0717-cg0723) deletion construct	this work
pK19*mobsacB*Δ*crtB2I’I2*	pK19*mobsacB* with a *crtB2I’I2* (cg2668-cg2672) deletion construct	this work

**Table 2:** Oligonucleotides used in this study.

Strain	DNA Sequence
*crtOP*-A	AAAACCCGGGTAGCTCCATATAACGTGCCG
*crtOP*-B	CCCATCCACTAAACTTAAACAGATTGTCATGCCATTGTCCAT
*crtOP*-C	TGTTTAAGTTTAGTGGATGGGACGATACTGCTAATAGCAATTCATCAGATATAA
*crtOP*-D	AAAACCCGGGATGTGTGGGAGGCTTCGC
*crtOP*-E	GTGACCATGAGGGCGAAAGC
*crtOP*-F	AAAACAATGCGCAGCGCA
*crtB2I’I2*-A	AAAACCCGGGGTCAGTGCTGTCATCGGTAC
*crtB2I’I2*-B	CCCATCCACTAAACTTAAACAATCTTGCTGATCAGCCAC
*crtB2I’I2*-C	TGTTTAAGTTTAGTGGATGGGAACAGTGTGGATCGGACTTAA
*crtB2I’I2*-D	AAAACCCGGGCTGCATGAATGTTGGTGAAC
*crtB2I’I2*-E	CGGACTTGATGCTGCAGC
*crtB2I’I2*-F	TGAGCCGCAACCAATTGAAG
*PcPS*-fw	*AACTGCCACACGAAC***GAAAGGAGGCCCTTCA**GATGGAGCTGTACGCCCAGAG
*PcPS*-rv	*GCATGCCTGCAGGTCGACTCTAGAGGATC*TTAGCCGCTGCCGTAGGG
*ispA*-fw	*ATGGAATTCGAGCTCGGTACCCGGG***GAAAGGAGGCCCTTCA**GATGGACTTTCCGCAGCAACTCG
*ispA*-rv	*GTTCGTGTGGCAGTT*TTATTTATTACGCTGGATGATGTAGTCC
*araBAD*-fw	*TGCAGGTCGACTCTAGAG***GAAAGGAGGCCCTTCA**GATGGCGATTGCAATTGGCCT
*araBAD*-rv	*GAGCTCGGTACCCGGGGATC*TTACTGCCCGTAATATGCCT
pEC-XT fw	AATACGCAAACCGCCTCTCC
pEC-XT rv	TACTGCCGCCAGGCAAATTC
Sequence in bold: artificial ribosome binding site; sequence in italics: linker sequence for hybridization.	

**Table 3:** Patchoulol production from 100 mM glucose in shake flasks (20 mL) after 48 h. Patchoulol titers and productivity refer to the total culture volume and are represented as mean values and standard deviations of biological triplicates. For comparison wild-type *C. glutamicum* grew to a biomass concentration of 5.4 g L^−1^ CDW from 100 mM glucose in the presence of dodecane [17].

	PAT1	PAT2	PAT3
CDW [g L^−1^]	4.4 ± 0.6	4.2 ± 0.6	4.2 ± 0.7
Titer [mg L^−1^]	0.20 ± 0.03	0.21 ± 0.02	0.46 ± 0.07
Vol. productivity [mg L^−1^ d^−1^]	0.10 ± 0.01	0.11 ± 0.01	0.23 ± 0.03

## Supplementary Materials

The following are available online at http://www.mdpi.com/2073-4425/9/4/219/s1, Figure S1: Phenotypes of patchoulol-overproducing PAT1 and PAT2 strains, Figure S2: GC-MS analysis of patchoulol.
