# Supplementary material for: Patchoulol Production with Metabolically Engineered Corynebacterium glutamicum
Source: Genes (Basel). 2018 Apr 17;9(4):219. doi: 10.3390/genes9040219 (PMC5924561; doi:10.3390/genes9040219)
Supplement: Supplementary file 1 [file genes-09-00219-s001.pdf]

Supplementary Material

Patchoulol production with metabolically engineered *Corynebacterium glutamicum*

Nadja A. Henke [1], Julian Wichmann [2], Thomas Baier [2], Jonas Frohwitter [1], Kyle J. Lauersen [2], Joe M. Risse [3], Petra Peters-Wendisch [1], Olaf Kruse [2] and Volker F. Wendisch [1,*]

[1] Genetics of Prokaryotes, Faculty of Biology & CeBiTec, Bielefeld University, Bielefeld D-33615, Germany;
[2] Algae Biotechnology & Bioenergy, Faculty of Biology & CeBiTec, Bielefeld University, Bielefeld D-33615, Germany;
[3] Fermentation Technology, Technical Faculty & CeBiTec, Bielefeld University, Bielefeld D-33615, Germany;
* Correspondence: Volker.wendisch@uni-bielefeld.de (V.F.W.); Tel.: +49-521-106-5611

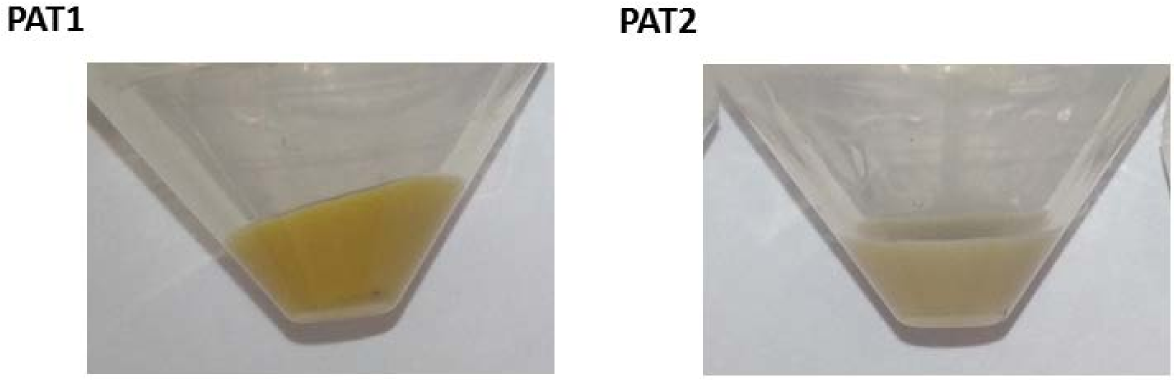

Figure S1: Phenotypes of patchoulol-overproducing PAT1 and PAT2 strains. The cell pellet of 10 mL of main culture is shown. PAT1: Δ*crtE*Δ*idsA* (pECXT-*ispA_Pc*PS)(pEKEx3); PAT2: Δ*crtOP*Δ*crtB2I'I2*Δ*idsA* (pECXT-*ispA_Pc*PS)(pVWEx1).

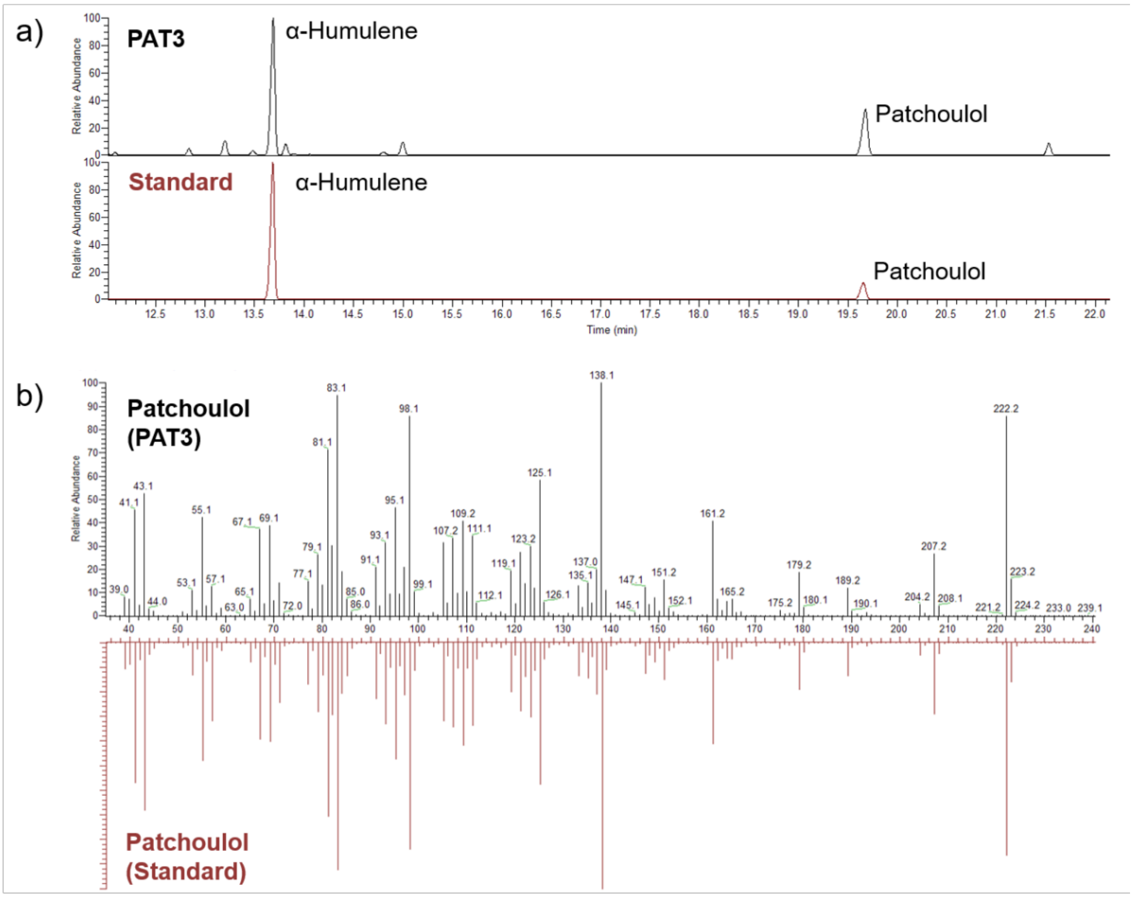

Figure S2: GC-MS analysis of patchoulol. a.) Extracted ion chromatogram (mass ranges 93.0, 138.50, 222.0) of PAT3 sample (black/ upper) and patchoulol standard (red/lower) supplemented with internal standard α-humulene. b.) Mass spectrum of patchoulol peak from PAT3 sample (black/upper) and patchoulol standard (red/lower).
